# The Book World of Medicine and Science

**Published:** 1895-05-25

**Authors:** 


### The Book World of Medicine and Science.

Anglo-Urdu Medical Handbook, or Hindustani Guide. For the use of Medical Practitioners (Male and Female) in Northern India. Compiled by the Rev. George Small, M.A., formerly Missionary at Benares, and latterly to Asiatics in London, in connection with the Strangers' Home, Limehouse; with the aid of Surgeon-General C. R. Francis, late of the Bengal Medical Service; and of Mrs. Fraser Nash, late Medical Missionary at Hyderabad, Scinde. (Thacker, Spink, and Co. Pp. 199.)

This book opens with a Hindustani vocabulary of the parts of the human body, followed by a vocabulary of diseases and of casualties. Among the latter we find delirium tremens, which surely is a disease. Next there are some forty-five pages on native medicines. In his preface, Surgeon-General Francis observes: "Medicines are annually brought from England to India at enormous cost," but a "wide field remains for the systematic and combined investigation of indigenous drugs." The researches, however, of Dimmock, King, Lall Dey, and others leave little to be discovered regarding the capabilities and virtues of Indian indigenous drugs. After the pages on native medicines there are the names of hospital and house furniture, followed by a "general medical vocabulary." Then we have short phrases in English and Hindustani. It is hoped that the book will be useful to English lady students preparing for India, to qualified medical missionaries, and to young surgeons of the Indian service, or connected with sea-going vessels to and from the East. Doubtless this will be the case. But the book being compiled for Northern India, various words used in the southerly districts are not mentioned.

How to Live in Tropical Africa: A Guide to Tropical Hygiene. The Malaria Problem: The Cause, Prevention, and Cure of Malarial Fevers. By J. Murray, M.D. Maps and Climatological Memoranda by E. G. Ravenstein, F.R.G.S. With Illustrations. (London: George Philip and Son, 32, Fleet Street, E.C. 1895. Pp. 252.)

Maculloch long since wrote, "Malaria produces in itself a far wider mass of human misery than any other cause of disease. It is the cause of far more than half the mortality of mankind." Dr. Murray, although not mentioning this opinion, certainly endorses it, for he says, "Impurities of the atmosphere from noxious emanations, save miasmatic, are not pertinent to this inquiry, and may consequently be neglected," and he styles malarial disease "the ubiquitous lethal fever" of Africa. But he also mentions that the pestilential littorals that formed almost insuperable barriers, destroying the lives and healths of adventurers, are now in process of annihilation by steam, by which a central highland region has been thrown open, presenting an inviting field for European expansion.

It must not, however, be supposed that even the central highland regions are free from malaria. For the author himself tells us that under such promising auspices the colonisa tion of Africa cannot be effected without a heavy tribute of sickness and mortality. And he gives much good advice as to the means by which malaria may be avoided.

Following in the footsteps of Marchiafava, Celli, Laverau, and Manson, Dr. Murray regards malaria as a parasitic micro-organism which destroys the blood corpuscles, and he regards quinine as the one specific and certain remedy. It may, however, be observed that, notwithstanding the researches of scientists, there are as yet various unanswered objections to the reception of the parasitic theory of malaria, and that a very short practice in the tropics will demonstrate that quinine is not quite the specific which it has been represented to be. Remarks on food, drink, and clothing follow, all of which are excellent. And we fully endorse the following advice: "If a man is not sound in wind and limb, if he has previously suffered from malarial fever, if he be scorbutic or anæmic, in God's name let him not go to tropical Africa."

